# Gastrointestinal mucormycosis with no classical immunosuppression and the potential for Qat leaves chewing as an unidentified risk factor: a case report

**DOI:** 10.3389/fmed.2026.1901474

**Published:** 2026-09-17

**Authors:** Fahad S. Alanazi, Hamad A. Alyami, Saeed R. Alzubide, Oweida F. Aldosary, Saleh Y. Alomar, Ali S. F. Owayed, Yahya A. Mohzari, Ahmad Alamer

**Affiliations:** 1 Department of Internal Medicine, King Saud Medical City, Riyadh, Saudi Arabia; 2 Department of Adult Infectious Diseases, King Saud Medical City, Riyadh, Saudi Arabia; 3 Department of Adult Gastroenterology, King Saud Medical City, Riyadh, Saudi Arabia; 4 Department of Anti-Microbial Stewardship, King Saud Medical City, Riyadh, Saudi Arabia; 5 Department of Clinical Pharmacy, College of Pharmacy, Prince Sattam bin Abdulaziz University, Alkharj, Saudi Arabia

**Keywords:** case report, diabetes mellitus, gastric mucormycosis, gastric ulcer, gastrointestinal (GI) bleeding, khat or qat (Catha edulis), mucormycosis

## Abstract

Gastrointestinal mucormycosis is a rare, life-threatening invasive fungal infection that is often diagnosed late because of nonspecific clinical and endoscopic findings. Khat or Qat (Catha edulis) leaves may harbor environmental fungi, including Mucor species, and chronic chewing may contribute to gastrointestinal mucosal injury. However, a possible association between Qat use and gastrointestinal mucormycosis has not been established. We report a case of a 60-year-old man with diet-controlled type 2 diabetes mellitus and long-standing Qat chewing who was admitted after an out-of-hospital cardiac arrest secondary to acute myocardial infarction. He had no history of malignancy, transplantation, neutropenia, or immunosuppressive therapy. Return of spontaneous circulation was achieved after 34 min of cardiopulmonary resuscitation, followed by temporary venoarterial extracorporeal membrane oxygenation. On hospital day 14, coffee-ground material was observed through the nasogastric tube, accompanied by a decline in hemoglobin from 14.0 to 9.5 g/dL. Esophagogastroduodenoscopy revealed a fungating, ulcerated lesion involving the gastric fundus and body. Histopathological examination demonstrated extensive necrosis, angioinvasion, and broad, ribbon-like, pauci-septate fungal hyphae with irregular branching, consistent with gastric mucormycosis. Intravenous liposomal amphotericin B was initiated at 8 mg/kg/day. Gastrointestinal bleeding resolved without surgery. Repeat endoscopy one month later showed marked healing, and follow-up biopsy revealed no residual fungal elements. The patient completed six weeks of antifungal therapy and achieved clinical and histopathological resolution. Fungal culture and molecular identification were not performed, precluding species-level confirmation.‏ In conclusion, this case describes gastric mucormycosis in a patient without profound immunosuppression but with severe physiological stress and chronic Qat exposure. Critical illness, gastric hypoperfusion, and mucosal disruption may have facilitated fungal invasion. Qat chewing is presented as a hypothesis-generating factor because of possible fungal contamination of leaves and repeated mucosal injury; nevertheless, causality cannot be inferred from a single case. Further microbiological and epidemiological studies are required to investigate this potential association.

## 1 Introduction

Mucormycosis is a life-threatening invasive fungal infection caused by fungi of the order *Mucorales* first described in 1885 by the Austrian pathologist Arnold Paltauf, who named it Mycosis Mucorina (1). Among members of the *Mucoraceae* family, *Rhizopus oryzae* (*Rhizopus arrhizus*) is the most commonly implicated species (2), typically found in animal feces and decaying organic matter. Other less frequently reported species include *Rhizopus microsporus var. rhizopodiformis*, *Absidia corymbifera*, *Apophysomyces elegans*, *Mucor* species, and *Rhizomucor pusillus* (3).

Mucormycosis predominantly affects immunocompromised individuals, particularly organ transplant recipients and patients with uncontrolled diabetes mellitus, and accounts for approximately 2% of invasive fungal infections in solid organ transplant populations (4). The disease is characterized by angioinvasion, resulting in vascular thrombosis, tissue necrosis, and potential dissemination (2, 5). Gastrointestinal mucormycosis is a rare manifestation, representing approximately 7%–13% of cases, with the stomach being the most involved site. Diagnosis is often delayed due to nonspecific clinical presentations such as abdominal pain, hematemesis, or melena, contributing to high mortality rates that may reach up to 85% (6). Although classically associated with profound immunosuppression, an increasing number of cases have been reported in patients without overt immunocompromising conditions, suggesting that transient physiological insults may also predispose to infection.

We report a case of gastric mucormycosis in a patient with well-controlled diabetes and no classical severe immunosuppression and explore the potential contributory role of chronic Qat (*Catha edulis*) chewing as a hypothesis-generating factor.

### 1.1 Case presentation

A 60-year-old male with a known history of type 2 diabetes mellitus, managed through dietary modification alone and demonstrating a baseline glycated hemoglobin of 6.5%, presented to the emergency department complaining of acute chest pain. The patient denied smoking. His medical history was further notable for habitual Qat (*Catha edulis*) chewing, a practice he had maintained for many years untill the time of presentation. Upon arrival, he experienced sudden cardiovascular collapse, prompting activation of the institutional code blue protocol. Cardiopulmonary resuscitation was initiated in accordance with Advanced Cardiovascular Life Support guidelines, and return of spontaneous circulation was achieved following 34 min of resuscitative efforts.

Given the patient's hemodynamic instability, the extracorporeal membrane oxygenation team was mobilized, and the patient was cannulated for venoarterial support. Electrocardiography demonstrated ST-segment elevation in the lateral leads, consistent with acute lateral wall myocardial infarction. Urgent coronary angiography identified a severe lesion in the distal left circumflex artery, which was treated with drug-coated balloon angioplasty. The subsequent clinical trajectory proved favorable; the patient was successfully decannulated from extracorporeal support on the fourth hospital day and transferred to the coronary care unit in stable condition.

On the fourteenth day of hospitalization, however, the patient developed coffee-ground drainage from his nasogastric tube. Laboratory evaluation revealed a substantial decline in hemoglobin concentration, from 14.0 g/dL on admission to 9.5 g/dL, raising concern for acute upper gastrointestinal hemorrhage. Esophagogastroduodenoscopy was performed, disclosing a medium-sized, fungating, and ulcerated noncircumferential mass within the gastric fundus and body, without active bleeding or stigmata of recent hemorrhage (Figure 1).

**Figure 1:**
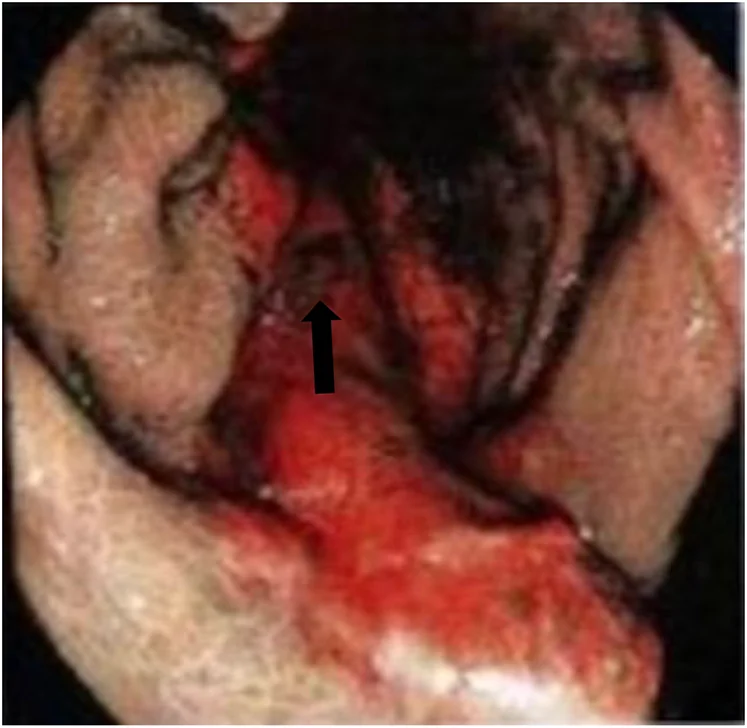
Endoscopic appearance of gastric mucormycosis at presentation. Esophagogastroduodenoscopy (EGD) demonstrating an irregular, ulcerated, non-circumferential lesion in the gastric fundus with a necrotic, mass-like appearance and no stigmata of active bleeding. The endoscopic morphology is consistent with necrotic inflammatory tissue rather than a true neoplasm, in keeping with angioinvasive fungal infection. Image quality is suboptimal as the procedure was performed at the bedside due to the patient's critical condition.

Biopsy specimens were obtained and submitted for histopathological examination. Microscopic evaluation of sections stained with Periodic Acid-Schiff revealed extensive areas of necrosis admixed with inflammatory exudate. Throughout the necrotic tissue and within vessel walls, numerous broad, ribbon-like, nonseptate fungal hyphae exhibiting irregular right-angle branching were identified (Figure 2), morphological features entirely consistent with *Mucorales* species. These findings established the diagnosis of gastric mucormycosis.

**Figure 2:**
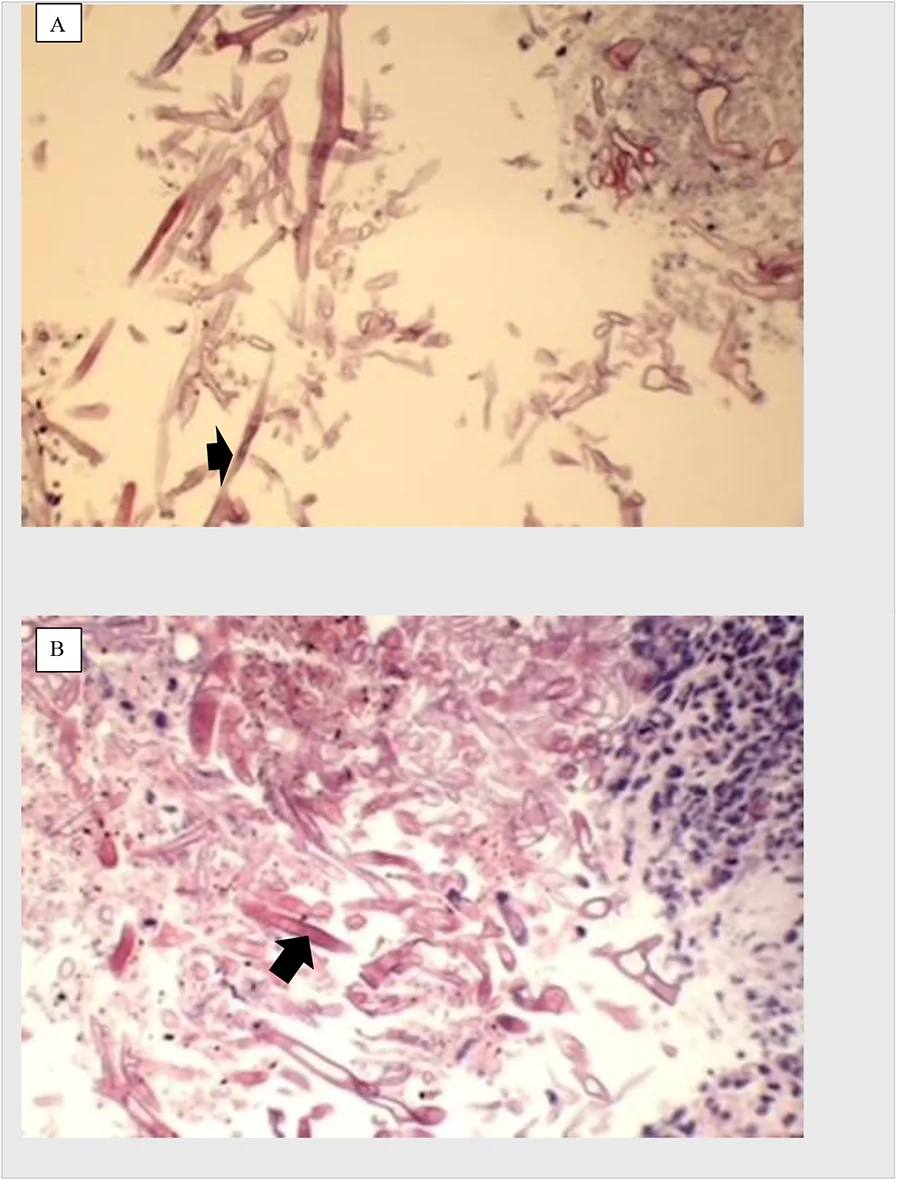
Histopathological features of gastric mucormycosis (PAS stain). Histopathological examination of gastric biopsy specimens stained with Periodic Acid–Schiff (PAS). **(A)** Low-power view demonstrating extensive tissue necrosis with associated inflammatory exudate. **(B)** High-power view showing broad, ribbon-like, pauciseptate fungal hyphae with irregular right-angle branching (arrows), infiltrating necrotic tissue and vessel walls. These findings confirm angioinvasion characteristic of Mucorales species.

### 1.2 Treatment and clinical course

Upon confirmation of the diagnosis, intravenous liposomal amphotericin B was initiated at a dose of 8 mg/kg daily. Computed tomography of the abdomen with intravenous was performed to evaluate for potential extension or complications, demonstrated a collapsed stomach without a definite fundal mass, perigastric fat stranding, or lymphadenopathy (Figure 3). Following the institution of antifungal therapy, the patient's gastrointestinal bleeding resolved, and no further episodes of hemorrhage occurred.

**Figure 3:**
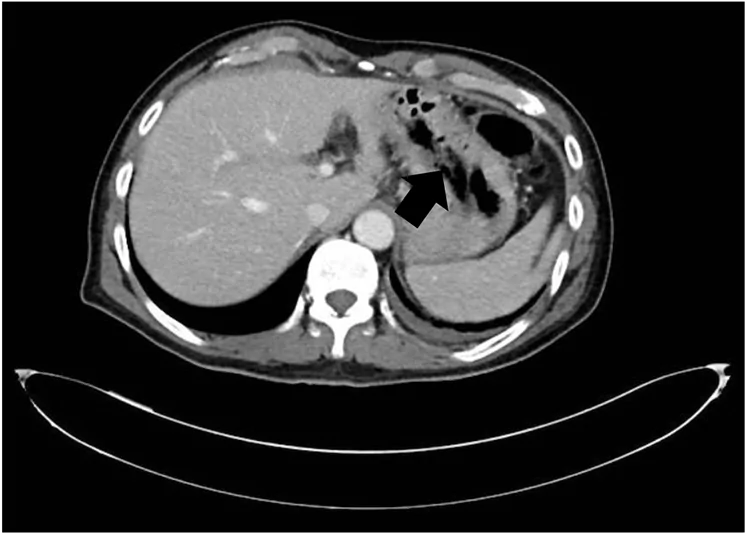
Computed tomography findings of the abdomen*.* Axial contrast-enhanced computed tomography (CT) image of the abdomen demonstrating a collapsed stomach without evidence of a discrete mass, perigastric fat stranding, or lymphadenopathy. The absence of a definable lesion on CT, despite the mass-like appearance on endoscopy, supports the interpretation of necrotic, non-neoplastic tissue.

Approximately one month following the index endoscopy, a repeat esophagogastroduodenoscopy was performed to assess treatment response. This examination revealed a 25-mm healed ulcer at the gastric fundus, with the scar tissue appearing healthy and demonstrating marked improvement compared with the initial endoscopic findings (Figure 4). Biopsy of the healed ulcer site demonstrated focal erosion with mild chronic inflammation; intestinal metaplasia, dysplasia, viral cytopathic changes, and fungal elements were notably absent on Hematoxylin and Eosin staining (Figure 5). The patient completed a six-week course of intravenous antifungal therapy, during which he demonstrated progressive clinical improvement. At the conclusion of treatment, he was alert and oriented with a Glasgow Coma Scale score of 15/15, was hemodynamically stable without further gastrointestinal bleeding, and had an improved hemoglobin concentration. Notably, surgical intervention was not required at any point during his clinical course.

**Figure 4:**
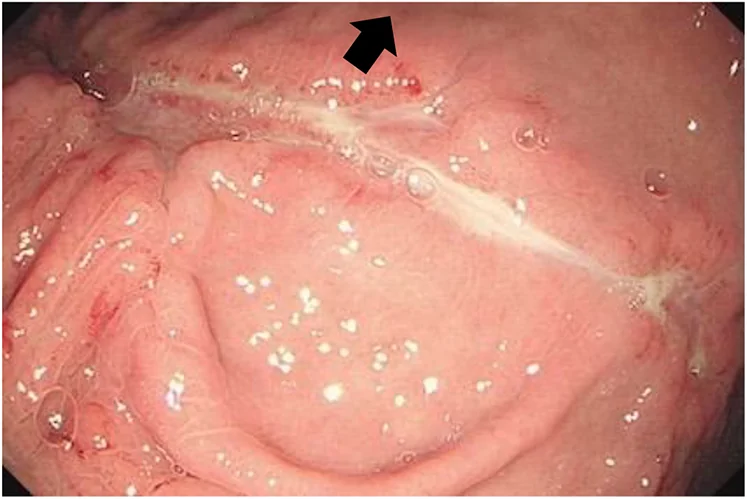
Endoscopic findings after antifungal therapy. Follow-up esophagogastroduodenoscopy performed one month after initiation of antifungal therapy, showing a healed ulcer in the gastric fundus with well-formed scar tissue and resolution of the previously observed necrotic mass-like lesion, indicating a favorable therapeutic response.

**Figure 5:**
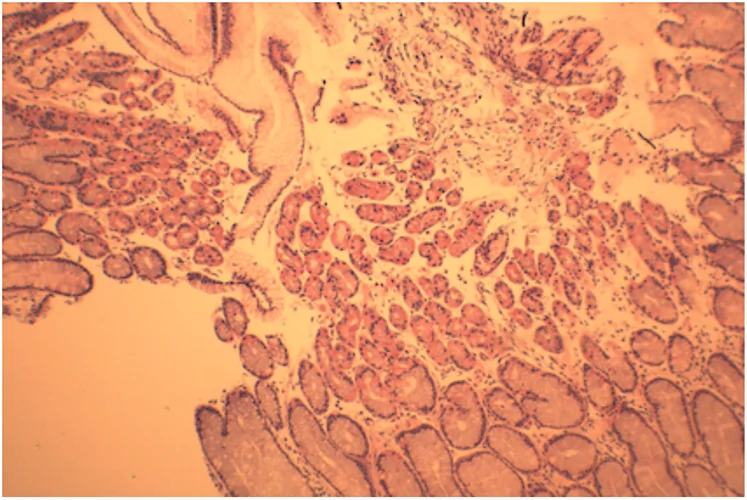
Histological resolution following treatment (H&E stain). Hematoxylin and eosin (H&E) staining of follow-up gastric biopsy demonstrating preserved gastric fundic mucosa with mild chronic inflammatory infiltrate. No fungal elements, intestinal metaplasia, dysplasia, or malignancy are identified, confirming histological resolution of infection.

## 2 Discussion

This case highlights the role of transient immune dysfunction and mucosal injury as potential enabling factors. The patient's recent myocardial infarction, prolonged cardiopulmonary resuscitation, extracorporeal membrane oxygenation (ECMO) support, and intensive care unit stay likely contributed to a multifactorial state of vulnerability. These conditions are associated with systemic inflammatory responses, impaired mucosal perfusion, ischemia-reperfusion injury, and disruption of the gastrointestinal barrier, all of which may facilitate fungal invasion (5). In addition, diabetes mellitus although well controlled in this patient, may have contributed by impairing neutrophil function, including chemotaxis and phagocytosis, thereby further reducing host defense mechanisms (2).

Mucormycosis is traditionally regarded as an opportunistic infection occurring in patients with significant immunosuppression, particularly those with uncontrolled diabetes mellitus, hematologic malignancies, or those receiving immunosuppressive therapy (3, 5). However, emerging evidence indicates that gastrointestinal mucormycosis may also occur in patients without classical severe immunosuppression, particularly in the setting of transient physiological stress. Gastrointestinal mucormycosis's epidemiology has evolved from predominantly affecting infants and premature neonates before 1990 and solid-organ transplant recipients during the 1990s to occurring in more diverse populations, including patients with diabetes, corticosteroid exposure, gastric ulcers, or no recognized predisposing factors. Because its clinical presentation is nonspecific, a high index of suspicion is warranted in immunosuppressed patients presenting with invasive gastrointestinal disease, with histopathological examination often needed to establish the diagnosis (7, 8). A literature review conducted by Kaur et al. covering the period between 1948 and 2017 in which the authors reviewed 176 cases of gastrointestinal mucormycosis in apparently immunocompetent patients. Majority of the reported cases were from Asia (50.6%), and the most affected anatomic sites were the intestine (64.2%) followed by stomach (33%) (7). Another periodic systematic review of case reports between 2015 and 2021 analyzed 70 adult cases (44% mortality) and 10 neonatal patients (70% mortality) with gastric mucormycosis. In 70% of adult cases, histopathological examination was used to establish the diagnosis, compared with 100% of neonatal cases. Only a few cases required histopathological examination and culture (11%), while an additional 7% required histopathological examination, culture, and polymerase chain reaction (PCR) to establish the clinical diagnosis. The authors of the periodic systematic review identified several risk factors in immunocompromised patients such as corticosteroid use, diabetes, blood cancers, organ transplantation, and alcohol use. Interestingly, 11% of the patients did not have classical immunosuppression or a clearly identifiable cause (8). Huang et al. reported two immunocompetent patients from China with no classical immunosuppression factors. In one interesting case, authors reported a 16-year-old male patient with no obvious immunosuppression who was admitted for gastrointestinal bleeding and perforation due to a gastric ulcer. Given the extent of the site of involvement, the patient underwent complete type II gastric resection. The postoperative pathological report suggested chronic gastroduodenal ulcer with necrosis, perforation, mycosis and complicated with bacterial infection (9).

A distinctive aspect of this case is the patient's long-standing Qat use (Catha edulis) contains several pharmacologically active compounds, of which cathinone (the most abundant), cathine, and cathedulines are considered the principal contributors to its pharmacological effects. It also contains tannins and small amounts of thiamine, niacin, riboflavin, iron, and amino acids. The gastrointestinal effects such as gastritis are due to the astringent tannins that irritate the gastric mucosa. When Qat leaves are freshly picked, cathinone (with a half life of 1.5 h), which resembles the chemical structure of D-(+) amphetamine, can exert its effect on the central nervous system (CNS). In addition to its CNS stimulant effect, cathinone may have peripheral effects such as dry mouth, blurred vision, mydriasis, increase in blood pressure, elevated heart rate, and maybe has an association with acute myocardial infarction (10). While Qat is well known for its sympathomimetic effects and association with cardiovascular complications (11, 12), its role in predisposing to invasive fungal infections has not been previously described. One of the earliest report of mycotoxin-producing potential of fungi associated with *Catha edulis* was by the biology department at the University of Sana'a, Yemen. In their 1991 publication, Al-Hubaishi et al. tested 24 varieties of Qat leaves in Yemen and identified 86 species belonging to 31 genera. The most frequent genera were *Cladosporium*, *Aspergillus*, and *Alternaria*, followed by *Penicillium*, *Drechslera*, *Fusarium*, *Curvularia*, *Phoma*, and *Chaetomium*. *Mucor* was also isolated, but its occurrence was low and represented 0.5%–0.7% of total fungi (13). Another study by A.-L.E. Mahmoud (2000) reported *Aspergillus, Alternaria, Penicillium*, and *Cladosporium,* followed by *Fusarium, Drechslera, Chaetomium*, and *Mucor* to be the most common fungal species found in Qat leaves (*Mucor hiemalis* and *Mucor racemosus*) (14).

Based on the available literature, several mechanisms may be hypothesized. First, Qat leaves are often consumed fresh and may be contaminated with environmental fungal spores, including *Mucorales*. Second, chronic chewing may cause repeated mucosal microtrauma, potentially facilitating microbial entry. Third, cathinone-induced vasoconstriction may impair mucosal blood flow, creating a hypoxic and acidic microenvironment favorable for fungal proliferation (15–17). Finally, chronic exposure may alter gastric mucosal integrity (18, 19). Among these, mucosal ischemia and direct environmental exposure appear the most biologically plausible mechanisms. However, in the absence of microbiological confirmation of fungal contamination, this association remains speculative and should be interpreted cautiously.

In previously reported cases, gastrointestinal mucormycosis is often associated with delayed diagnosis and poor outcomes, frequently requiring surgical intervention (20, 21). In contrast, this patient demonstrated complete clinical and histological resolution with antifungal therapy alone, without the need for surgery. This favorable outcome may be attributable to early diagnosis and prompt initiation of treatment. We note that the differential diagnosis includes gastrointestinal basidiobolomycosis, particularly in patients without classical immunosuppression. However, the prominent angioinvasion with associated thrombosis and extensive tissue necrosis favors mucormycosis. Gastrointestinal basidiobolomycosis more typically demonstrates eosinophil-rich granulomatous inflammation, fibrosis, and Splendore–Hoeppli material surrounding the fungal hyphae. Nevertheless, rare cases of angioinvasive basidiobolomycosis have been reported; therefore, in the absence of culture or molecular identification, these findings are supportive of, but not entirely specific for, mucormycosis (22–26). The histopathological differential diagnosis should also include *Aspergillus, Scedosporium, and Fusarium* species (26). *Candida* species should also be considered when yeast forms with pseudohyphae are observed (28). Moreover, establishing a diagnosis of mucormycosis based solely on histopathological examination can be challenging, particularly because Mucorales may be misidentified as *Aspergillus spp*., which represents the most common diagnostic errors. In terms of histopathology, *Mucorales* hyphae have a variable width of 6–16 μm and can go up to 25 μm, and are nonseptate or pauci-septate (28). In specific tissues, the hyphae can appear ribbon-like with an irregular pattern of branching (see the histopathological description of our case Figure 2). In contrast, *Aspergillus* hyphae are typically narrow and regularly septate, with dichotomous branching. Therefore, we acknowledge a major limitation of the present report is the absence of fungal culture or molecular identification, which precluded confirmation at the genus or species level. In fact, the joint initiative of the European Confederation of Medical Mycology and the Mycoses Study Group Education and Research Consortium acknowledges the current limitations of histopathological diagnosis. Although immunohistochemical techniques using commercially available monoclonal antibodies and polymerase chain reaction–based methods have demonstrated high specificity, their reported sensitivity remains variable. Current recommendations continue to support the identification of *Mucorales* hyphae based on their characteristic morphology, while recognizing the limited evidence regarding the accuracy of differentiating *Mucorales* from other moulds by histopathology alone. Furthermore, obtaining cultures from clinical samples is highly encouraged to allow determination of the organism at both the genus and species levels. Molecular approaches are likewise strongly advocated and are favored over methods relying solely on morphological features, with sequencing of the internal transcribed spacer region considered the preferred technique. The use of matrix-assisted laser desorption/ionization time-of-flight mass spectrometry receives moderate support, as its effectiveness is largely influenced by the availability and quality of local or institutional reference databases, which may not be present in all settings (26).

Determining the organism at the genus and species levels is strongly advised to enhance epidemiological insights into mucormycosis, while relying on genus-level identification alone to inform treatment decisions is only weakly supported. In a similar vein, routine antifungal susceptibility testing for *Mucorales* may offer clinical value in instances of therapeutic failure; however, the evidence supporting its regular use in guiding treatment remains limited. Consequently, such testing is primarily recommended for epidemiological purposes and for advancing understanding of antifungal resistance patterns (26).

Prompt recognition and early initiation of antifungal therapy are critical to improving outcomes in mucormycosis. Amphotericin B remains the cornerstone of treatment and is recommended as first-line therapy in current international guidelines, often combined with surgical debridement when feasible (26). Overall, this case supports the concept that mucormycosis may occur in the setting of combined moderate risk factors and transient physiological stress, rather than requiring profound immunosuppression alone, and raises the potential habitual use of *Cathis edulis* as a suspected risk factor that requires further investigation.

## 3 Conclusions

This case highlights the importance of considering mucormycosis in the differential diagnosis of gastrointestinal bleeding, even in patients without classical severe immunosuppression. The combination of diabetes mellitus, acute critical illness, and possible environmental exposure may act synergistically to facilitate infection. Chronic Qat use may represent a potential contributing factor; however, this association remains speculative and requires further investigation. Early recognition and prompt initiation of antifungal therapy are essential and may allow successful treatment without surgical intervention in selected cases.

## Data Availability

The original contributions presented in the study are included in the article/Supplementary Material, further inquiries can be directed to the corresponding author.
